# Extensive subpial cortical demyelination is specific to multiple sclerosis

**DOI:** 10.1111/bpa.12813

**Published:** 2020-02-03

**Authors:** Andreas Junker, Jadwiga Wozniak, David Voigt, Uta Scheidt, Jack Antel, Christiane Wegner, Wolfgang Brück, Christine Stadelmann

**Affiliations:** ^1^ Institute of Neuropathology University Medical Center Göttingen Georg August University Göttingen Robert‐Koch‐Str. 40 37075 Göttingen Germany; ^2^ Department of Neuropathology University Hospital Essen Hufelandstr. 55 45147 Essen Germany; ^3^ Montreal Neurological Institute McGill University Health Centre 2155 Guy Street Montreal Canada; ^4^ Department of Child and Adolescent Psychiatry/Psychotherapy University Medical Center Göttingen Georg August University Göttingen Robert‐Koch‐Str. 40 37075 Göttingen Germany

**Keywords:** multiple sclerosis (MS), progressive multifocal leukoencephalopathy (PML), cortical demyelination, subpial lesions, meningitis, carcinomatous and lymphomatous meningitis, neuromyelitis optica (NMO), acute disseminated encephalomyelitis (ADEM), oxidative stress

## Abstract

Cortical demyelinated lesions are frequent and widespread in chronic multiple sclerosis (MS) patients, and may contribute to disease progression. Inflammation and related oxidative stress have been proposed as central mediators of cortical damage, yet meningeal and cortical inflammation is not specific to MS, but also occurs in other diseases. The first aim of this study was to test whether cortical demyelination was specific for demyelinating CNS diseases compared to other CNS disorders with prominent meningeal and cortical inflammation. The second aim was to assess whether oxidative tissue damage was associated with the extent of neuroaxonal damage. We studied a large cohort of patients diagnosed with demyelinating CNS diseases and non‐demyelinating diseases of autoimmune, infectious, neoplastic or metabolic origin affecting the meninges and the cortex. Included were patients with MS, acute disseminated encephalomyelitis (ADEM), neuromyelitis optica (NMO), viral and bacterial meningoencephalitis, progressive multifocal leukoencephalopathy (PML), subacute sclerosing panencephalitis (SSPE), carcinomatous and lymphomatous meningitis and metabolic disorders such as extrapontine myelinolysis, thus encompassing a wide range of adaptive and innate cytokine signatures. Using myelin protein immunohistochemistry, we found cortical demyelination in MS, ADEM, PML and extrapontine myelinolysis, whereby each condition showed a disease‐specific histopathological pattern. Remarkably, extensive ribbon‐like subpial demyelination was only observed in MS, thus providing an important pathogenetic and diagnostic cue. Cortical oxidative injury was detected in both demyelinating and non‐demyelinating CNS disorders. Our data demonstrate that meningeal and cortical inflammation alone accompanied by oxidative stress are not sufficient to generate the extensive subpial cortical demyelination found in MS, but require other MS‐specific factors.

## Introduction

Cortical demyelinated lesions are observed in over 90% of patients with chronic MS in post‐mortem neuropathological studies 4. In extreme cases, up to 70% of the cortical area was found to be demyelinated, and in a proportion of patients, the percentage of demyelinated cortex exceeded that of white matter lesions. This identifies the cerebral cortex as a predilection site for tissue damage in MS 43. Distinct types of cortical MS lesions have been defined according to their location 13, 14: Type I (cortico‐subcortical or leukocortical) lesions are located at the border between gray and white matter and extend into both; type II lesions are found within the gray matter (intracortical lesions) and are often formed around small vessels; and type III subpial lesions form ribbon‐like areas of demyelination in the upper cortical layers and frequently extend over neighboring gyri and sulci 34, 43. Subpial demyelinated lesions comprise the largest fraction (65%) of cortical lesions 14. Although cortical demyelinated lesions already occur early in the disease, extensive areas of cortical demyelination were observed in patients with chronic disease, thus identifying cortical demyelination as a pathological correlate of disease progression 4, 43, 46.

Cortical demyelination may contribute to neuronal and synaptic dysfunction and loss 23, 75, although this loss is likely to correspond to the structural correlate of cortical atrophy 50. In imaging studies, cortical atrophy is strongly correlated with non‐focal symptoms in MS such as cognitive decline, and considered to be part of the neurodegenerative features of the disease 16. Cortical atrophy, and therefore the severity of cortical damage, might therefore serve as a robust early predictor of progressive disease 24, 27.

The pathophysiological mechanisms leading to cortical demyelination still remain enigmatic 68. Myelin and oligodendrocyte damage may be brought on by diverse mechanisms such as an antigen‐specific humoral or cellular immune response, a bystander effect of toxic cytokines, genetic/metabolic disturbances, viral infection or ischemic and excitotoxic damage 17, 28. In the case of MS, it has been proposed that immune‐mediated demyelination is precipitated by an antibody/complement attack 71, the presence of pro‐inflammatory cytokines 29, oxidative damage 45 or by cytotoxic T cells 57. In several studies, subpial cortical demyelination has been linked to meningeal inflammatory infiltration, in particular the number of T and B cells 20, 34, 35, 49. Also, Ig transcripts were detected in molecular studies of the MS cortex, highlighting the presence of B and plasma cells in the meninges 73. The extent of meningeal inflammation correlated with shorter survival and a more severe disease course 34. MRI demonstrated focal leptomeningeal contrast enhancement in 25% of investigated MS cases. Corresponding pathology showed an inflammatory infiltration in association with subpial demyelination 1. A gradient of cortical neuronal loss was also found, further linking meningeal inflammation to neurodegeneration 48. It therefore seems plausible that meningeal inflammatory infiltrates, and possibly also the secreted mediators thereof, contribute to both cortical demyelination and MS disease progression.

In accordance, on the molecular level an upregulation of adaptive and innate inflammatory as well as cell death and tissue repair genes have also been reported and it was shown that the combination of inflammation, plaque‐like primary demyelination and neurodegeneration in the cortex is specific to multiple sclerosis and not seen in other chronic inflammatory diseases 26. Moreover, oxidative damage to neurons was found to be characteristic of MS when compared to other chronic (meningeal) inflammatory diseases such as tuberculosis and Alzheimer's disease. Microglial expression of NADPH oxidase (p22phox) was observed here in close proximity to neurons containing oxidized phospholipids 26. Mitochondrial gene deletions in cortical neurons of SPMS patients have also been found, potentially resulting from chronic oxidative damage 18.

In the present study, we examined a large cohort of autoimmune, inflammatory and neoplastic diseases of the central nervous system (CNS) with potentially predominant affection of the meninges and upper cortical layers. We hypothesized that among the diseases studied, the inflammatory milieu might in part overlap with that found in MS. Therefore, similar immune effector pathways may be activated, leading to damage of the underlying cortical elements such as myelin, oligodendrocytes, neurons and axons. Cases with acute as well as long‐standing meningeal inflammation were included. However, immunohistochemical assessment using antibodies against myelin proteins failed to identify extensive subpial demyelination in our cohort of non‐MS patients. Subpial demyelination was only identified in MS autopsies and a subset of PML cases with high local viral load, whereby the subpial demyelination pattern was extensive in MS and focal in PML. Oxidative damage in cortical lesions was associated with neuronal injury but not necessarily with demyelination, and was identified in PML, extrapontine myelinolysis (EPM), HSV encephalitis and SSPE. Our findings indicate that neither acute granulocytic nor chronic lymphocytic meningeal inflammation alone is sufficient to selectively damage myelin and oligodendroglia and lead to the typical subpial demyelination found in MS.

## Materials and Methods

### Human brain tissue

This study was performed on formalin‐fixed, paraffin‐embedded (FFPE) archival brain tissue from 156 cases (155 autopsy cases and 1 biopsy case) with a variety of demyelinating (inflammatory and non‐inflammatory metabolic) as well as non‐demyelinating (inflammatory and neoplastic) CNS diseases (Table 1 and Supporting Table S2) obtained from the archives of the Institute of Neuropathology, University Medical Center Göttingen, Germany, and the Institute of Neurology, McGill University, Montreal, Canada. To study intracortical changes in acute PML, we also included one biopsy case with cortical JC virus load. In total, 457 archival tissue blocks were investigated, and the diagnoses were categorized into 19 disease groups (Table 1). Tissue blocks derived from the frontal, parietal, temporal and occipital cortex. Cortical demyelinated MS lesions from the cohort were analyzed in detail in a previous study 4. The MS patients had a mean disease duration of 17 years (mean ± SD: 17.8 ± 6.4, median: 20.5 years). The neuropathological diagnosis in all patients was confirmed by three neuropathologists (WB, CS and AJ). In addition, the clinical history and the results of whole body autopsy, where available, were reviewed. All investigations were performed in compliance with relevant laws and institutional guidelines, and were approved by the local ethics committee.

**Table 1:** Neuropathological entities investigated

Category	Diagnosis	Number of cases	Number of cortical tissue blocks examined
Demyelinating CNS diseases			
Autoimmune	Multiple sclerosis	33	180
Autoimmune	ADEM	3	14
Autoimmune	NMO	6	21
Viral	JC virus (PML)	11	39
Metabolic	Central pontine myelinolysis/extrapontine myelinolysis	6	14
Non‐demyelinating CNS diseases			
Neoplastic	Non‐Hodgkin lymphoma	18	30
Neoplastic	Hodgkin lymphoma	3	4
Neoplastic	Plasmocytoma	3	5
Neoplastic	Carcinomatous meningitis	11	20
Viral	HIV	2	4
Viral	Subacute sclerosing panencephalitis (SSPE)	2	7
Viral	Poliomyelitis	5	10
Viral	Viral meningitis/meningoencephalitis with defined pathogen (not including HIV, measles virus, poliovirus or JC virus)	8	16
Bacterial	Tuberculosis	13	22
Bacterial	Syphilis	3	16
Bacterial	Bacterial meningitis with defined pathogen (not including tuberculosis or syphilis)	12	20
Infectious without identified pathogen	Acute *lymphocytic* meningitis/meningoencephalitis without defined pathogen	12	25
Infectious without identified pathogen	Acute *granulocytic* meningitis/meningoencephalitis without defined pathogen	5	10
	Total number of disease entities: 19	Total number of cases: 156	Total number of tissue blocks: 457

### Histology and immunohistochemistry

Histological evaluation was performed on 3‐µm‐thick sections stained with HE and Luxol fast blue (LFB)‐PAS to assess inflammation and demyelination. Axons were stained with Bielschowsky's silver impregnation. Proof of Gram‐positive bacteria was made on the basis of Gram staining. Ziehl–Neelsen staining demonstrated the presence of acid‐fast mycobacteria. Chloroacetate esterase enzyme histochemistry was performed to visualize granulocytes. Immunohistochemistry (IHC) was performed after antigen‐unmasking microwave treatment for 15 min (800 W) in citrate or EDTA buffer. Endogenous peroxidase activity was blocked by incubation of the sections in 3% H_2_O_2_ in PBS. Sections were blocked with 10% fetal calf serum in PBS for 10 min at room temperature. Washed sections were stained with the antibodies listed in Supporting Table S1. Bound antibody was visualized using an avidin–biotin technique with 3, 3′‐diaminobenzidine (DAB) or 3‐amino‐9‐ethylcarbazole (AEC) as chromogens.

### Tissue and lesion classification

All blocks underwent detailed neuropathological examination and were screened for gray and white matter myelin damage. As it is difficult to detect gray matter lesions (GML) on sections stained with LFB‐PAS, we performed proteolipid protein (PLP), myelin basic protein (MBP), myelin oligodendrocyte glycoprotein (MOG) and myelin‐associated glycoprotein (MAG) IHC to visualize cortical myelin sheaths. The number and location of demyelinated lesions (in MS and PML tissue blocks), the composition of the inflammatory infiltrate (granulocytes, lymphocytes, macrophages), and the presence of meningeal inflammation and cortical involvement were recorded. Graphs were generated using GraphPad PRISM (PRISM 5.0, GraphPad, San Diego, CA). The severity of neoplastic or inflammatory meningeal infiltrations of non‐autoimmune diseases was assessed as follows: 0 = no meningeal infiltration; 1 = sparse infiltration (less than 50 infiltrating cells per high‐power field = 400x); 2 = moderate infiltration (more than 50 and less than 200 infiltrating cells per high‐power field); 3 = dense infiltration (more than 200 infiltrating cells per high‐power field).

## Results

### Cortical demyelination is present in MS and other demyelinating CNS diseases

**Figure 1:**
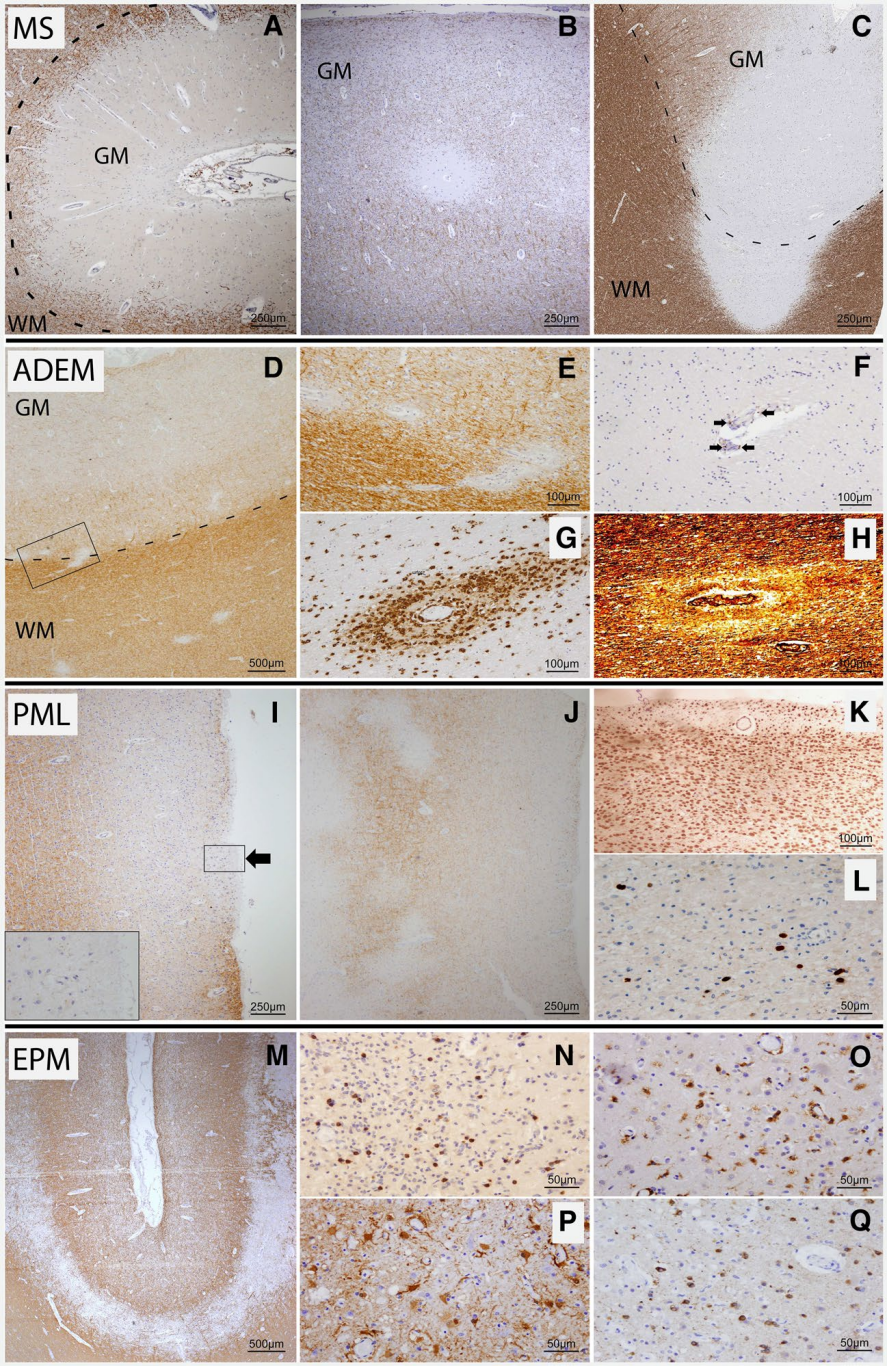
*Distinct features of cortical demyelination in classical demyelinating diseases*. Cortical MS lesions are depicted in **A–C** (MPB IHC). **A** shows a subpial, ribbon‐like demyelinated lesion involving the upper cortical layers of neighboring gyri (type III lesion). **B** displays a well‐delineated, small perivenous intracortical (type II) lesion. In **C,** a leukocortical (type I) lesion involving both cortical gray and white matter is shown (WM = white matter; GM = gray matter). **D–H** show leukocortical demyelination in ADEM. Small perivascular demyelinated foci can be identified in the deep cortex by MBP IHC (**D** and **E** (higher magnification of area delineated in **D**)). No subpial demyelination was observed. Few perivascular CD3^+^ T lymphocytes (arrows) are depicted in **F.** Foamy perivascular macrophages and activated microglial cells are highlighted in **G** using KiM1P IHC. **H** shows the preserved but loosened axonal scaffold in Bielschowsky's silver impregnation. Cortical demyelination with subpial demyelinated areas in PML is shown in **I**–**L**. Small, irregular demyelinated subpial foci can be identified in serial sections in MBP IHC (**I**). **J** (higher magnification of area delineated in **I**) shows demyelinated intracortical and leukocortical lesions in MBP IHC (same patient as **I**, different cortical area). **K** shows a subpial demyelinated area full of foamy macrophages that is highlighted with KiM1P IHC (same lesion as in **I**). The high viral load of this area is shown in panel **L** (SV40 IHC). The ribbon‐like cortico‐subcortical demyelination detected in one patient with extrapontine myelinolysis (EPM) is shown in **M–Q**. Cortico‐subcortical demyelination spanning adjacent gyri (PLP‐IHC). A dense infiltration by CD8^+^ T cells (**N**) and KiM1P^+^ macrophages/activated microglia (**O**) is found in the demyelinated area. **P** shows the intense reactive astrogliosis (GFAP IHC). Nogo A^+^ oligodendrocytes are abundant in the demyelinated area (**Q**).

Cortical demyelinated lesions were detected in 94% (29 of 33) of chronic MS patients in our cohort, as described previously 4. We characterized cortical lesions identified by MBP, PLP or MOG IHC into three types: *type I* lesions encompassed both white and gray matter (Figure 1C), *type II* lesions were found in the gray matter surrounding microvessels (Figure 1B) and *type III* lesions were subpial and in part ribbon like (Figure 1A). Focal meningeal lymphocytic aggregates were not consistently observed adjacent to type III lesions. However, the meninges were not preserved in all cases, so our assessment had to remain incomplete. Also, the subpial demyelinated lesions investigated here showed only a sparse infiltration of lymphocytes 4.

In acute disseminated encephalomyelitis (ADEM), perivenous demyelination in the white matter was apparent by IHC for myelin proteins (Figure 1D–H). Perivenous demyelination was also observed at the white–gray matter junction (Figure 1E) and occasionally intracortically. However, subpial demyelination as seen in MS could not be identified in the cases studied here.

Although neuromyelitis optica (NMO) can resemble MS clinically and pathologically, myelin and oligodendrocytes are not the primary target of the immune response. In our cohort of patients, no evidence of cortical and especially subpial demyelination was observed (data not shown). In addition, no evidence of subpial astrocyte loss or microglia activation was found in the cases studied here 31.

Typical white matter lesions in our cases with PML (Figure 1I–L) contained foamy macrophages, only few lymphocytes—mainly CD8^+^—and astrocytes with bizarre cell body and nuclear shapes. Oligodendrocytes in part displayed ground‐glass nuclei and were reduced in number. JC virus infection was confirmed with SV40 immunoreactivity in the cytoplasm and nucleus of several oligodendrocytes in the lesions (Figure 1L). Leukocortical lesions were present in 7/10 autopsy cases with PML. Intracortical demyelinated lesions with fuzzy borders occurred in 6/10 autopsy cases; 1 additional biopsy case studied showed intracortical and subpial lesions. 2/10 autopsy cases also showed subpial demyelinated areas whereby these 2 cases displayed prominent cortical inflammation and intense cortical virus load. However, in contrast to MS, the subpial demyelinated areas observed in PML were focal, small, fairly round and without sharp borders (Figure 1I). Subpial myelin was completely absent in these areas and foamy phagocytes containing myelin debris were abundant in the lesions. Axons were relatively preserved but many APP^+^ spheroids marked the acute axonal damage. We found an exceptionally high virus load in these upper cortical lesions. Astrocytes showed a high degree of nuclear polymorphism, and oligodendrocytes were substantially reduced in these lesions.

In one of the six patients diagnosed with central pontine myelinolysis (CPM) (Table 1) who presented with typical pontine lesions, we found extrapontine demyelinated areas with myelin loss and preserved axonal structures (extrapontine myelinolysis—EPM) (Figure 1M–Q). These lesions affected predominantly the cortex and partly the underlying subcortical white matter. According to the lesion typing applied to MS cortical pathology, these lesions resembled type I MS lesions 59. They contained abundant foamy macrophages with a considerable number of mainly CD8^+^ T cells.

### Preserved cortical myelin in non‐demyelinating CNS diseases

To assess the effect of meningeal and cortical inflammation on the underlying cortical elements such as myelin, we examined other CNS disorders with prominent meningeal and cortical inflammation (Supporting Figure S1 and Table S2).

**Figure 2:**
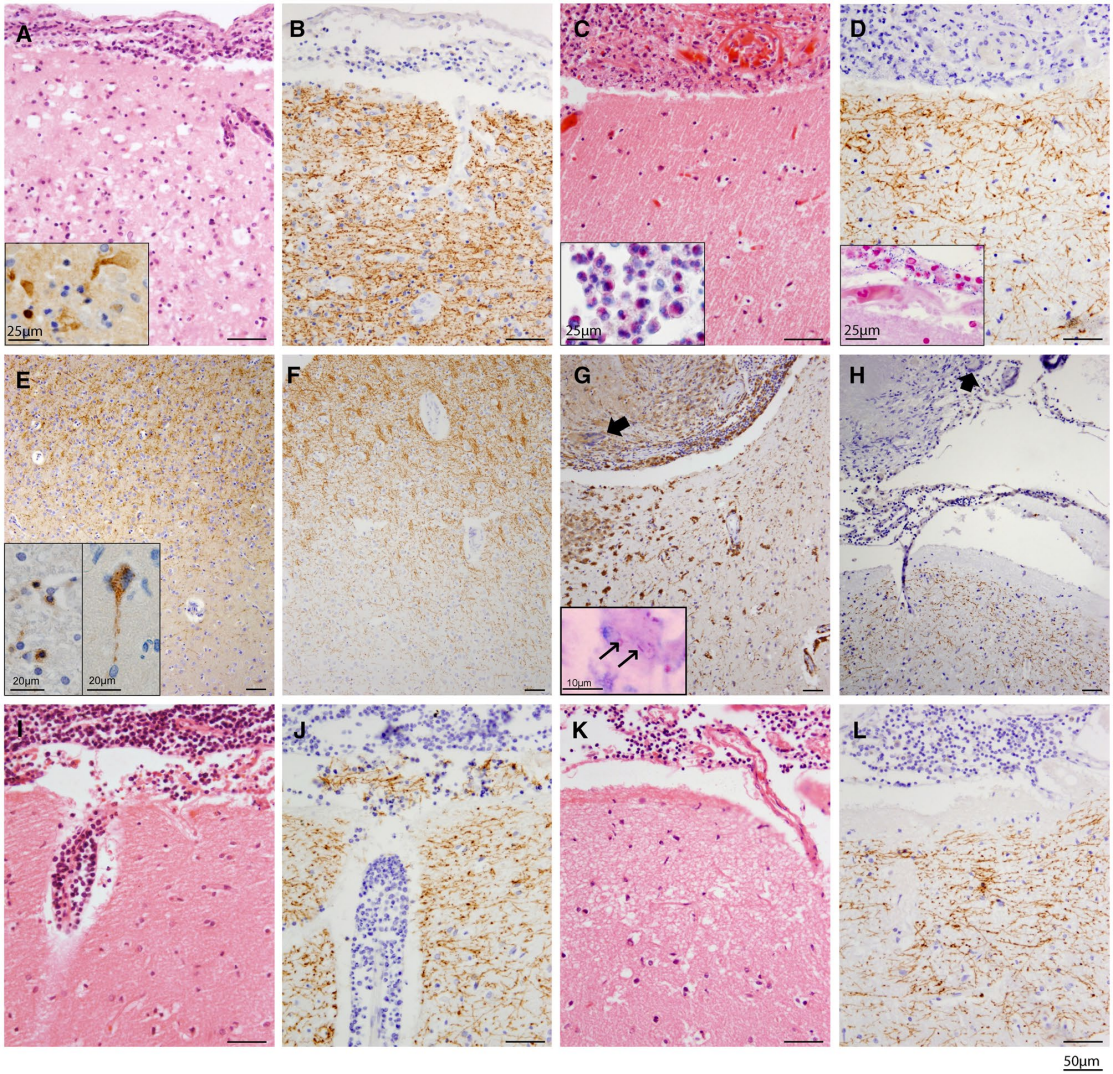
*Diseases with meningeal and/or cortical pathology without cortical demyelination*. HE (**A, C**) and IHC for PLP (**B, D**) of cortex with adjacent meningeal inflammatory infiltration shows preserved subpial myelin sheaths in HSV encephalitis (**A, B**) and acute bacterial meningitis (**C, D**). The inset in **A** depicts HSV1^+^ neurons. Chloroacetate esterase^+^ granulocytes are shown as inset in **C**, and Gram^+^ bacteria in **D**. No evidence of demyelination was found when applying IHC for MAG (**E**) and PLP (**F**)— neither in the white nor gray matter—in a representative case of measles virus‐induced subacute sclerosing panencephalitis (SSPE). Measles virus antigen is detected by IHC in oligodendrocytes and neurons (insets in **E**). Meningeal infiltration with lymphocytes, epitheloid cells and giant cells (arrows in **G** and **H**) as well as caseous necrosis characterizes tuberculosis (**G**, KiM1P IHC). Acid‐fast mycobacteria are visualized by Ziehl–Neelsen staining (inset). PLP IHC demonstrates preserved subpial myelin immediately adjacent to a meningeal tuberculoma (**H**). HE (**I, K**) and IHC for PLP (**J, L**) of cortex with adjacent meningeal infiltration shows preserved subpial myelin sheaths in lymphomatous meningitis (**I, J**) and plasmocytoma (**K, L**).

*Acute viral meningitis* was characterized by a predominance of mononuclear infiltrates in the meninges. We studied several patients with HSV meningoencephalitis (Figure 2A,B). In these cases, cortical necrosis and infiltration of PMNs were observed, and HSV in neurons was detected by IHC. We found moderate numbers of lymphocytes and macrophages extending from the pial surface through the cerebral cortex into the subcortical white matter. No cortical demyelination was seen. Cases with cytomegalovirus infection showed pathological changes exclusively in the white matter, where we found cytomegalic inclusion cells, ependymitis and tissue necrosis. We could not detect any specific damage to myelin or oligodendrocytes either in these cases or in cases with acute lymphocytic meningitis without defined pathogen.

Cases diagnosed with *acute bacterial meningitis* showed an extensive meningeal infiltration with granulocytes, macrophages and few lymphocytes. In most cases (>90%), focal cortical areas were also affected by inflammatory infiltrates, and about 50% of the cases showed focal cortical necrosis associated with inflammatory infiltrates. In patients who died at later disease stages, meningeal infiltration was dominated by lymphocytes and macrophages with few granulocytes. We did not observe any demyelination in these cases (Figure 2C,D).

We also studied tissue specimens from two patients who died from subacute sclerosing panencephalitis (SSPE) with proven measles virus infection (Figure 2E,F). Sparse infiltration of T lymphocytes with massive microglial activation displaying numerous microglial nodules throughout the cortical and white matter was observed. Intracytoplasmic or nucleic inclusions as well as measles virus antigen were detected in many oligodendrocytes. IHC for myelin proteins did not show any evidence of demyelination in the gray or white matter either in these cases or in cases with non‐specific plasma and B cell‐rich meningeal inflammation, where no pathogen could be determined.

*Syphilis* and *tuberculosis* are chronic infections with treponema pallidum and mycobacterium ssp., respectively. The bacteria survive phagocytosis by macrophages and elicit a strong delayed‐type hypersensitivity response. Both diseases can cause granulomas, characterized by necrosis, epitheloid and giant cells and lymphocytic infiltration. The current concept of granuloma and giant cell formation favors several cytokines such as IFNγ and TNFα as causative inflammatory mediators 19, 63. The leptomeninges are a major site of involvement in granulomatous diseases 76. In one of the three syphilis cases examined, we found several ischemic white and gray matter lesions. However, no primary demyelinating lesions were observed; similarly, no damage to the subpial myelin sheaths was found in any of the cortical areas examined. In 10 of the 13 cases with tuberculosis studied, we found a significant meningeal infiltration with lymphocytes and macrophages. Meningeal granulomata were found in 4 of the 13 investigated cases (Figure 2G,H). However, no cortical and particularly no subpial demyelination was observed.

Demyelination has been described in the context of CNS non‐Hodgkin lymphoma (NHL) 5, 9, 36, 42. In our cohort of 18 NHL patients (Table 1), 11 showed cortical or meningeal infiltration; of our 3 patients with Hodgkin lymphoma, 1 harbored cortical and meningeal infiltrates (Supporting Figure S1 and Table S2). However, no demyelination was observed in either gray or white matter in any of the lymphoma patients (Figure 2I,J).

Chronic meningeal, plasma cell‐rich inflammation has been related to subpial demyelination in MS 34, 48. However, our cases with meningeal plasmocytoma infiltration did not show any cortical demyelination (Figure 2K,L). We also investigated 11 cases of meningeal carcinomatosis where malignant cells had infiltrated the subarachnoid space diffusely or in glandular formations. Our collection contained specimens of bronchial carcinoma, ovarian carcinoma, breast cancer and gastrointestinal carcinoma. Subpial cortical layers contained few activated astrocytes, activated microglial cells and hardly any lymphocytic infiltration. The subpial myelin layers appeared intact and did not show myelin loss in any of the investigated cases.

### Evidence of cortical oxidative injury in inflammatory CNS diseases with and without demyelination

**Figure 3:**
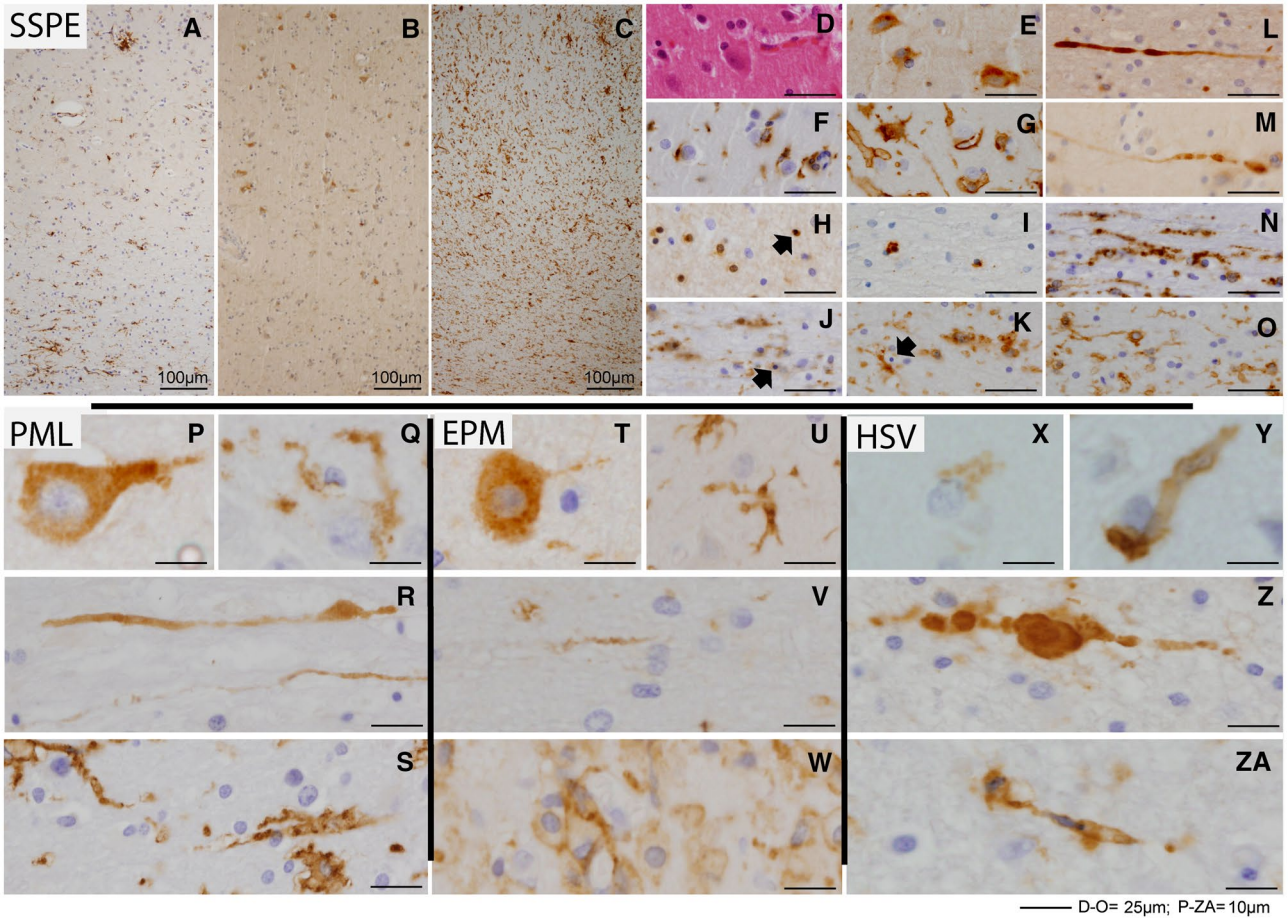
*Oxidative damage in cortical lesions in PML, EPM, HSV and SSPE is associated with neuronal injury*. Extensive microglia activation in the gray and white matter with microglia nodules is observed in a representative case of measles virus‐induced subacute sclerosing panencephalitis (SSPE) (**A**). Numerous cells with strong cytoplasmic E06 immunoreactivity (oxidized phospholipids) are seen in a corresponding section in the cortex (**B**). A corresponding section of **B** shows massive expression of the NADPH oxidase component p22phox in microglia cells within the cortex but also in the underlying white matter (**C**). A higher magnification of cortical neurons (**D** HE) with strong cytoplasmic E06 immunoreactivity (**E**) shows their close contact with numerous KiM1P^+^ (**F**) and p22phox^+^ (**G**) microglia; in the staining against p22phox, single small vessels are additionally marked positively. Oligodendrocytes in the white matter are shown in **H** (p25 IHC). In our samples, no demyelination was observed in SSPE in either the gray or the white matter. However, scattered apoptotic oligodendrocytes were visible, particularly in the white matter (arrows in **H, J**). Measles virus antigen was detected by IHC in many oligodendrocytes in the white matter (**I**). Apoptotic and virus‐infected oligodendrocytes were surrounded by activated microglia cells (KiM1P IHC in **J**) with high expression of the NADPH oxidase component p22phox (**K**). **L** shows a fragmented, damaged axon (APP IHC). Fragmented, damaged axons in the white matter of SSPE accumulated oxidized phospholipids (E06 reactivity in **M**) and were seen in close proximity to KiM1P^+^ microglia cells (**N**) which were positive for the NADPH oxidase component p22phox (**O**). Similarly, we identified damaged neurons with strong cytoplasmic E06 immunoreactivity (oxidized phospholipids) also in PML (**P**), EPM (**T**) and HSV encephalitis (**X**). Moreover, these entities showed fragmented axons in the white matter with strong E06 immunoreactivity (**R** in PML, **V** in EPM, **Z** in HSV encephalitis). P22phox^+^‐activated microglia surrounded oxidatively damaged neurons (**Q** in PML, **U** in EPM or **Y** in HSV encephalitis) or were observed in the vicinity of fragmented axons (APP^+^) in these diseases (**S** in PML, **W** in EPM and **ZA** in HSV encephalitis).

Cortical demyelination is likely to be caused by disease‐specific factors. In MS lesions, oxidative tissue damage was suggested to be a major driving force behind demyelination and tissue damage 25. Causative agents such as reactive oxygen species (ROS) are synthesized by special enzyme systems including myeloperoxidase (MPO) or nicotinamide adenine dinucleotide phosphate (NADPH) oxidase in activated microglia and macrophages 25, 30, 51. The extent of lipid and DNA oxidation correlated with inflammation (and oxidative stress) in MS lesions 32. As oxidative stress in MS lesions has already been studied before, our study focused on non‐MS inflammatory and demyelinating conditions to examine oxidative stress with regard to the expression of p22phox, a subunit of NADPH oxidase and the occurrence of oxidized phospholipids (E06) as a marker for oxidative tissue injury. We found numerous microglial cells to be immunoreactive for p22phox, with a staining pattern resembling that of the pan‐macrophage marker KiM1P in SSPE, PML, EPM, HSV encephalitis, TBC and ADEM. A particularly high density of p22phox‐positive microglia cells was found in the cortex and white matter of patients with SSPE (Figure 3A–O). In the cortex, SSPE cases showed many neurons with a strong cytoplasmic immunoreactivity for oxidized phospholipids which were surrounded by p22phox‐positive microglia cells. In the white matter microglial cells were scattered between morphologically intact myelin sheaths. High immunoreactivity for oxidized phospholipids was also detected in axonal spheroids. Apart from SSPE (Figure 3A–O), we could also localize oxidized phospholipids to degenerating axons and neurons in PML (Figure 3P–S), EPM (Figure 3T–W) and HSV encephalitis (Figure 3X–ZA). Neurons in part showed the morphological features of retrograde chromatolysis. Furthermore, we found similar changes in a low number of neurons and axons in meningeal tuberculosis cases and ADEM (data not shown). Interestingly, in PML and EPM, neurons with oxidized phospholipids frequently were seen in cortical areas adjacent to cortical/subcortical demyelinated lesions, again with signs of retrograde chromatolysis. In summary oxidative injury was present in inflammatory CNS disorders with and without demyelination.

## Discussion

### Ribbon‐like, extensive subpial demyelination is specific to MS

Cortical demyelination and in particular extensive subpial myelin loss is characteristic of MS. Other classical demyelinating diseases such as ADEM 77 and PML 56 as well as diseases with acute and chronic inflammatory and neoplastic cortical pathology could potentially also feature cortical or subpial myelin alterations. Therefore, in this large‐scale study, we further investigated whether the subpial and cortical pathology of MS is unique to this disease. In addition, we elucidated whether oxidative stress, a hallmark of the pathogenesis of demyelination and tissue injury in MS 8, 11, 12, 25, 32, 33, 65, 66, 74, occurred in other inflammatory or demyelinating diseases. We thus analyzed diverse disease conditions with a wide range of adaptive and innate immune cell infiltration and patterns of cytokine secretion. Tissue specimens of diseases other than MS such as PML, ADEM, NMO and CPM/EPM, where demyelination is a disease‐specific feature, were also included in our study. In PML brain tissue intracortical and leukocortical but not subpial lesions were observed before 56.

Of note, we observed leukocortical, intracortical *and subpial* demyelination in patients with PML. In contrast to MS, subpial cortical demyelinated lesions were invariably focal, small and roundish and appeared in cortical areas with a large viral load. ADEM patients exhibited only occasionally small perivenous leukocortical lesions. Perivenous subpial demyelination has been described in the literature in three cases with ADEM 77. One of our investigated cases with CPM/EPM showed extensive ribbon‐like leukocortical demyelinated lesions. However, we did not detect any evidence of ribbon‐like subpial myelin damage in any other disease than MS.

Although we have investigated a wide range of diseases with meningeal and cortical pathology in this study, no patients, for example, with anti‐MOG disease, have been included. We can thus not formally exclude that in the future, diseases from the inflammatory demyelinating spectrum will be identified, which, like classical multiple sclerosis, show ribbon‐like subpial demyelination.

### Cellular contribution to subpial demyelination in MS

Subpial cortical demyelination in MS has been associated with lymphocytic meningeal inflammation 49, 64. Inflammatory mediators released from meningeal inflammatory cells may contribute to cortical demyelination in MS 29, 48. Consistent with an activation of B and plasma cells, an upregulation of Ig molecules has been observed in MS cortex 73. However, a close association of inflammatory cells with the underlying demyelinated cortex has not been found consistently 40, suggesting that a more global inflammatory reaction—arguably present in the CSF—may be required for demyelination 67. Indeed, we recently demonstrated in a mouse model of cortical demyelination that—in addition to conformation‐specific, anti‐myelin antibodies—peripheral immune cells including CCR2^+^ monocytes are crucial for demyelination 44. In this study, we explicitly examined archival autopsy cases with meningeal and cortical pathology and specifically scrutinized cortical regions immediately adjacent to meningeal inflammatory and neoplastic cells for myelin pathology and oxidative stress.

### Reactive oxygen species and cytokines form disease‐specific milieus in demyelinating and non‐demyelinating CNS diseases

Oxidized lipids and oxidized DNA have been identified biochemically in brain tissue from MS patients 11, 61, 65, 74. Tissue injury caused by oxidative damage was described in inflammatory MS lesions in which oxidized phospholipids accumulated in dystrophic axons, degenerating neurons and damaged oligodendrocytes 32.

We detected oxidized lipids and NADPH oxidase (visualized with the marker p22phox, a subunit of NADPH oxidase), responsible for the generation of reactive oxygen species, in inflammatory non‐demyelinating diseases other than MS, such as SSPE and HSV encephalitis, as well as demyelinating diseases such as PML and EPM. In PML and EPM, neurons with oxidized phospholipids were often detected in areas adjacent to cortical/subcortical demyelinated lesions, a finding described earlier in MS 26. This suggests that disease‐specific mechanisms of demyelination are at work in MS which exceed common mechanisms of oxidative tissue damage. Candidates include specific combinations of cytokines and chemokines, which essentially determine the inflammatory tissue microenvironment.

MS lesions contain a complex cytokine/chemokine milieu. Pro‐inflammatory cytokines such as TNFα, IL‐1β and IFNγ mediate the acute inflammatory process in early MS lesions 6, 10, 58. Furthermore, active and chronic MS lesions show a considerable expression of TGFβ 21. The fact that cytokines may contribute to the development of cortical demyelination is also supported by the targeted cortical EAE models in which a stereotactic intracortical injection of TNFα and IFNγ in MOG‐immunized mice, rats and marmosets leads to focal cortical demyelinated lesions 44, 54, 69. Here, inflammatory cytokines contribute to an opening of the blood–brain barrier, allowing the influx of immune cells and soluble pro‐inflammatory mediators such as anti‐myelin antibodies and, for example, fibrinogen. Also, the diffuse neuronal and synaptic damage observed in the human disease and in experimental models suggests a role of diffusible, wide‐ranging pro‐inflammatory cytokines in cortical tissue damage in MS 3, 22, 39, 47, 55.

Our cohort included patients with acute, early inflammatory disease and non‐acute lymphocytic meningeal infiltration such as in chronic leptospiral and aseptic meningitis. Interestingly, several of these diseases show an overlap in cytokine/chemokine expression with MS. IL1β and TNFα are cytokines which are upregulated in the CSF in acute inflammatory CNS diseases such as bacterial and mycobacterial (IL1β, TNFα) or viral meningitis/meningoencephalitis (IL1β) 2. TGFβ is upregulated in reactive astrocytes in acute diseases such as bacterial meningoencephalitis 7, HSV encephalitis or PML 38. Other homeostatic chemokines such as CXCL12, CXCL13 or CCL19 and inflammatory cytokines such as CXCL10, CCL2 and CCL3 are involved in immune cell trafficking into diseased CNS tissue in MS 52. CXCL13 in combination with IL10 is upregulated in primary CNS lymphoma, and in this combination is specific for the disease 62. CNS lymphoma, especially when treated with steroids, may present as demyelination, suggesting that components released from dying lymphoma cells as well as the strong inflammatory micro‐milieu may induce myelin damage 9. However, none of our patients with a lymphoid neoplasm exhibited cortical or white matter myelin damage. Some metastatic brain tumors also show an upregulation of cytokines such as CXCL13 62. Nevertheless, none of our investigated cases with carcinoma metastases showed myelin damage adjacent to tumor cell infiltration.

None of the tissue specimens investigated here exhibited ribbon‐like subpial cortical demyelination, although the inflammatory mediators released by inflammatory and neoplastic CNS diseases as well as the extent of oxidative damage affecting the gray matter may overlap with MS. Thus, our studies suggest that it is not merely the level and combination of cytokines and chemokines that determines the occurrence of (subpial) cortical demyelination, but that additional factors come into play.

### The genetic background as a determinant of susceptibility to demyelination

In experimental models of MS, spontaneous cortical demyelination occurs in MOG‐immunized non‐human primates and selected rat strains 44, 53, 70. Targeted EAE models in which the focal injection of cytokines targets inflammatory cells and antibodies to a defined cortical area, allow for the precise localization and timing of cortical demyelination 54. The rare EAE models in which spontaneous cortical lesions develop suggest that genetic factors may be important in determining lesion location. Genes of the MHC locus that most likely influence microglia activation were shown to predispose certain rat strains to the formation of cortical demyelination 72. Noteworthy, the presence of anti‐MOG antibodies and proinflammatory cytokines such as tumor necrosis factor α (TNFα) and interferon γ (IFN γ) in the subarachnoid space seems to be sufficient to induce subpial cortical demyelination 29. In the marmoset EAE model in which intracortical and subpial lesions occur regularly early in disease, extensive subpial demyelination was related to meningeal T and plasma cell, but not B cell infiltration 41, 54, 60. The strong influence of the genetic background on demyelination is also obvious in experimental infection of mice with Theiler's murine encephalomyelitis virus (TMEV) which produces an acute polioencephalitis. Animals may recover but remain persistently infected and develop demyelination. Interestingly, TMEV infection produces demyelination in SJL/J mice but not in C57/Bl6 mice, where the virus is completely cleared 15.

The predominant inflammatory and potentially demyelinating milieu in the lesion is thus determined by the individual genetic background. Gene expression naturally varies according to the cell type. Thus, the manifestation of the genetic background depends decisively on the cellular composition of the lesion. Besides immune cells, also glial cells and neurons contribute to this specific microenvironment 37, 78, thus contributing to the extent of tissue damage in the individual patient.

In conclusion, our data indicate that widespread and ribbon‐like subpial cortical demyelination is a disease‐specific feature of MS. Even a wide array of adaptive and innate immune signatures reflected in the diseases studied here does not lead to subpial cortical demyelination. However, the genetic composure of an individual may strongly impact his or her propensity to develop cortical myelin pathology.

## Conflicts of Interest

The authors declare that they have no conflict of interest.

## Supporting information


**Figure S1. Semiquantitative assessment of cellular meningeal infiltration of non‐autoimmune diseases.** Cellular meningeal infiltration (neoplastic or inflammatory) was assessed semi‐quantitatively using four different categories: no infiltrating cells (0), little meningeal infiltration (less than 50 infiltrating cells per high power field (HPF) = 400x magnification) (1), moderate cellular meningeal infiltration (more than 50 and less than 200 infiltrating cells per high power field) (2) and dense cellular meningeal infiltration (more than 200 infiltrating cells per high power field) (3). The mean value of the meningeal infiltration per case is shown. The most severe inflammatory meningeal infiltrations were seen in cases with viral or bacterial meningitis and meningoencephalitis.
**Table S1.** Antibodies and staining procedures.
**Table S2.** Overview of autopsy cases* studied.

## Data Availability

The data that support the findings of this study are available from the corresponding author upon reasonable request.
