# Supplementary material for: Extensive subpial cortical demyelination is specific to multiple sclerosis
Source: Brain Pathol. 2020 Feb 3;30(3):641–52. doi: 10.1111/bpa.12813 (PMC8018087; doi:10.1111/bpa.12813)
Supplement: Supplementary file 1 — Figure S1. Semiquantitative assessment of cellular meningeal infiltration of non‐autoimmune diseases. Cellular meningeal infiltration (neoplastic or inflammatory) was assessed semi‐quantitatively using four different categories: no infiltrating cells (0), little meningeal infiltration (less than 50 infiltrating cells per high power field (HPF) = 400x magnification) (1), moderate cellular meningeal infiltration (more than 50 and less than 200 infiltrating cells per high power field) (2) and dense cellular meningeal infiltration (more than 200 infiltrating cells per high power field) (3). The mean value of the meningeal infiltration per case is shown. The most severe inflammatory meningeal infiltrations were seen in cases with viral or bacterial meningitis and meningoencephalitis. Table S1. Antibodies and staining procedures. Table S2. Overview of autopsy cases* studied. [file BPA-30-641-s001.docx]

**Extensive subpial cortical demyelination is specific to multiple sclerosis**

**Supplementary material:**

**Supplementary Figures:**

**
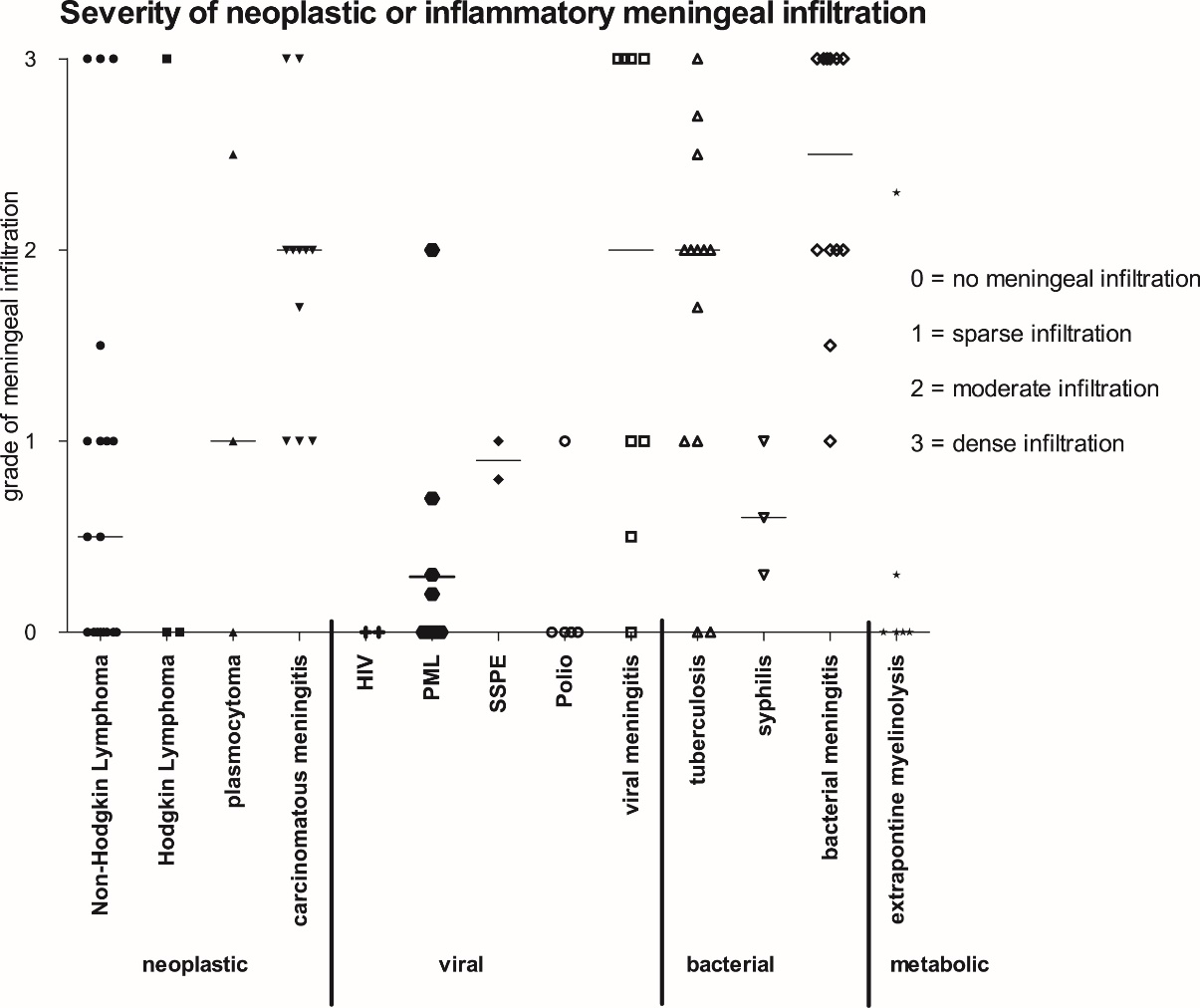
**

**Supplementary Figure 1: Semiquantitative assessment of cellular meningeal infiltration of non-autoimmune diseases**

Cellular meningeal infiltration (neoplastic or inflammatory) was assessed semi-quantitatively using four different categories: no infiltrating cells (0), little meningeal infiltration (less than 50 infiltrating cells per high power field (HPF) = 400x magnification) (1), moderate cellular meningeal infiltration (more than 50 and less than 200 infiltrating cells per high power field) (2) and dense cellular meningeal infiltration (more than 200 infiltrating cells per high power field) (3). The mean value of the meningeal infiltration per case is shown. The most severe inflammatory meningeal infiltrations were seen in cases with viral or bacterial meningitis and meningoencephalitis.

**Supplementary tables:**

| **Supplementary table 1**: antibodies and staining procedures | | | | |
| --- | --- | --- | --- | --- |
| **Antigen** | **Antibody  species** | **Dilution** | **Pretreatment** | **Company** |
| APP | mouse mc, MAB348 | 1:2000 | citrate | Chemicon |
| MBP | rabbit pc  REF A0623 | 1:2000 |  | DAKO |
| PLP | mouse mc  clone plpc1 | 1:500 | citrate | AbD Serotec |
| MAG | mouse mc | 1:10 | citrate | Hybridoma product |
| MOG | rat pc | 1:1000 | citrate | kind gift from Prof. D. Merkler, University of Geneva, Switzerland |
| CD 8 | mouse mc  clone C8/144B | 1:50 | citrate | DAKO |
| Pan macrophage KiM1P | mouse mc | 1:5000 | citrate | kind gift from Prof. HJ Radzun,  University of Göttingen, Germany |
| GFAP | rabbit pc  REF 0334 | 1:1000 |  | DAKO |
| Nogo A | rabbit pc | 1:1000 | citrate | Santa Cruz |
| NF 200 | mouse mc  clone 52 | 1:400 | citrate | Sigma |
| P25 | Rabbit pc  REF 92305 | 1:100 | EDTA | AbD Serotec |
| HSV1 | rabbit pc  REF 0114 | 1:500 | citrate | DAKO |
| Measles virus nucleocapsid | mouse mc  clone 2B4 | 1:500 | citrate | kind gift from Prof. JL Bennett,  University of Colorado, Denver USA |
| CD 20 | mouse mc  clone C26 | 1:100 |  | DAKO |
| CD 138 | mouse mc  clone MI15 | 1:100 | EDTA | DAKO |
| SV40 | mouse mc  clone Pab416 | 1:100 | citrate | Calbiochem |
| E06 | Mouse mc | 1:400 | citrate | Avanti |
| P22phox | Rabbit pc | 1:100 | citrate | Santa Cruz |

| **^Supplementary table 2:^**^Overview of autopsy cases* studied^ | | | | | | | | |
| --- | --- | --- | --- | --- | --- | --- | --- | --- |
| **Category of brain disease** | **Subcategory of brain disease** | **Diagnoses and concomitant diseases** | sex | **age**  **in years** | **disease duration** | **cause of death** | **number of blocks (with cortex)** | **^1^ severity of meningeal infiltration** |
| neoplastic | Non-Hodgkin Lymphoma, | meningeosis lymphomatosa; malignant B cell non Hodgkin lymphoma | m | 67 | 6 months | disease-related | 1(1) | **3.0** |
| neoplastic | Non-Hodgkin Lymphoma, | EBV-associated B cell non Hodgkin lymphoma | f | 71 | n.a. | disease-related | 1(1) | **0.0** |
| neoplastic | Non-Hodgkin Lymphoma, | meningeosis lymphomatosa; malignant B cell non Hodgkin lymphoma | f | 26 | 5 months | disease-related | 2(2) | **0.5** |
| neoplastic | Non-Hodgkin Lymphoma, | malignant B cell non Hodgkin lymphoma; acute cardiovascular failure | m | 53 | 1 year | disease-related | 3(3) | **0.0** |
| neoplastic | Non-Hodgkin Lymphoma, | malignant B cell non Hodgkin lymphoma; pulmonary edema; renal failure | f | 50 | 11 months | disease-related | 3(3) | **0.0** |
| neoplastic | Non-Hodgkin Lymphoma, | malignant B cell non Hodgkin lymphoma | f | 57 | n.a. | disease-related | 3(3) | **0.0** |
| neoplastic | Non-Hodgkin Lymphoma, | malignant B cell non Hodgkin lymphoma | f | 77 | 1 year | disease-related | 1(1) | **1.0** |
| neoplastic | Non-Hodgkin Lymphoma, | malignant B cell non Hodgkin lymphoma; pneumonia; septic shock | m | 39 | n.a. | disease-related | 1(1) | **0.0** |
| neoplastic | Non-Hodgkin Lymphoma, | follicular lymphoma; feverish condition | f | 57 | 2 years | not disease-related | 3(3) | **0.0** |
| neoplastic | Non-Hodgkin Lymphoma, | T cell non Hodgkin lymphoma; respiratory failure | m | 69 | 4 years | disease-related | 2(2) | **0.0** |
| neoplastic | Non-Hodgkin Lymphoma, | meningeal infiltration of chronic lymphatic leukemia | f | 66 | 1 year | disease-related | 1(1) | **1.0** |
| neoplastic | Non-Hodgkin Lymphoma, | meningeal infiltration of chronic lymphatic leukemia; pneumonia | f | 72 | 1.5 years | not disease-related | 1(1) | **1.0** |
| neoplastic | Non-Hodgkin Lymphoma, | acute lymphatic leukemia; acute cardiovascular failure | f | 69 | 20 days | disease-related | 1(1) | **0.0** |
| neoplastic | Non-Hodgkin Lymphoma, | meningeal infiltration of chronic lymphatic leukemia; pneumonia; acute cardiovascular failure | m | 75 | 15 months | disease-related | 2(2) | **0.5** |
| neoplastic | Non-Hodgkin Lymphoma, | acute myeloic leukemia | f | 53 | 6 months | disease-related | 2(2) | **1.5** |
| neoplastic | Non-Hodgkin Lymphoma, | acute undifferentiated leukemia; meningeosis leukemica | m | 12 | n.a. | disease-related | 1(1) | **3.0** |
| neoplastic | Non-Hodgkin Lymphoma, | meningeal infiltration of chronic myeloic leukemia; pneumonia | m | 71 | 8 weeks | disease-related | 1(1) | **1.0** |
| neoplastic | Non-Hodgkin Lymphoma, | acute myeloic leukemia | m | 0.3 | n.a. | disease-related | 1(1) | **3.0** |
|  |  |  |  |  |  |  |  |  |
| neoplastic | Hodgkin Lymphoma | Hodgkin Lymphoma | f | 18 | 1 year | disease-related | 1(1) | **0.0** |
| neoplastic | Hodgkin Lymphoma | Hodgkin Lymphoma | f | 34 | n.a. | disease-related | 1(1) | **0.0** |
| neoplastic | Hodgkin Lymphoma | Hodgkin Lymphoma | m | 71 | 2 months | disease-related | 2(2) | **3.0** |
|  |  |  |  |  |  |  |  |  |
| neoplastic | plasmocytoma | plasmocytoma; heart failure | f | 66 | 9 years | not disease-related | 2(2) | **2.5** |
| neoplastic | plasmocytoma | plasmocytoma; heart failure | f | 61 | 6 months | disease-related | 2(2) | **1.0** |
| neoplastic | plasmocytoma | plasmocytoma | m | 42 | 6 months | disease-related | 1(1) | **0.0** |
|  |  |  |  |  |  |  |  |  |
| neoplastic | carcinomatous meningitis | renal cell carcinoma and esophageal cancer | m | 63 | 2 years | disease-related | 2(2) | **2.0** |
| neoplastic | carcinomatous meningitis | Pancoast tumor | m | 48 | 8 months | disease-related | 1(1) | **3.0** |
| neoplastic | carcinomatous meningitis | breast cancer | f | 42 | n.a. | disease-related | 1(1) | **1.0** |
| neoplastic | carcinomatous meningitis | malignant mixed mesodermal tumor | f | 84 | n.a. | disease-related | 1(1) | **2.0** |
| neoplastic | carcinomatous meningitis | bronchial carcinoma | m | 43 | 3 years | disease-related | 1(1) | **3.0** |
| neoplastic | carcinomatous meningitis | carcinomatous meningitis; breast cancer | f | 64 | 6 months | disease-related | 2(2) | **2.0** |
| neoplastic | carcinomatous meningitis | carcinomatous meningitis; breast cancer | f | 34 | 7 months | disease-related | 3(3) | **2.0** |
| neoplastic | carcinomatous meningitis | carcinomatous meningitis; esophageal cancer | m | 60 | 18 months | disease-related | 3(3) | **1.7** |
| neoplastic | carcinomatous meningitis | Signet ring cell carcinoma | f | 76 | 1 month | disease-related | 2(2) | **2.0** |
| neoplastic | carcinomatous meningitis | carcinomatous meningitis; breast cancer | f | 41 | 5 years | disease-related | 2(2) | **1.0** |
| neoplastic | carcinomatous meningitis | carcinomatous meningitis; ovarian carcinoma | f | 74 | 9 years | disease-related | 2(2) | **1.0** |
|  |  |  |  |  |  |  |  |  |
| viral infections | HIV | HIV infection; pneumonia; Kaposi's sarcoma; toxoplasmosis | m | 34 | 5 years | disease-related | 2(2) | **0.0** |
| viral infections | HIV | HIV infection | f | 37 | n.a. | n.a. | 2(2) | **0.0** |
|  |  |  |  |  |  |  |  |  |
| viral infections | JC virus | PML | f | 59 | biopsy case | disease-related | 2(2) | **0.0** |
| viral infections | JC virus | PML | f | 78 | n.a. | disease-related | 4(4) | **0.0** |
| viral infections | JC virus | PML | m | 27 | 4 weeks | disease-related | 3(3) | **0.3** |
| viral infections | JC virus | PML | m | 45 | 3.5 months | disease-related | 4(4) | **0.0** |
| viral infections | JC virus | PML; HIV infection | m | 51 | 2 months | disease-related | 5(5) | **0.0** |
| viral infections | JC virus | PML after chemotherapy; oropharyngeal cancer | f | 58 | n.a. | disease-related | 4(4) | **0.0** |
| viral infections | JC virus | PML; HIV infection | m | 36 | n.a. | disease-related | 6(6) | **0.0** |
| viral infections | JC virus | PML; long term glucocorticoid treatment | m | 55 | 2 months | disease-related | 5(5) | **0.2** |
| viral infections | JC virus | PML; HIV infection | f | 40 | n.a. | disease-related | 2(2) | **0.0** |
| viral infections | JC virus | PML; chronic lymphocytic leukemia; aspergillosis pneumonia | m | 63 | n.a. | n.a. | 3(3) | **0.7** |
| viral infections | JC virus | PML; HIV infection; heroin addiction; | m | 33 | n.a. | n.a. | 1(1) | **2.0** |
|  |  |  |  |  |  |  |  |  |
| viral infections | subacute sclerosing panencephalitis (SSPE) | subacute sclerosing panencephalitis (SSPE); status post measles infection | m | 10 | 3 months | disease-related | 2(2) | **1.0** |
| viral infections | subacute sclerosing panencephalitis (SSPE) | subacute sclerosing panencephalitis (SSPE); status post measles infection; cardiac insufficiency; venous congestion | m | 8 | n.a. | disease-related | 5(5) | **0.8** |
|  |  |  |  |  |  |  |  |  |
| viral infections | poliomyelitis | poliomyelitis; polioencephalitis | m | 1.7 | 7 days | disease-related | 1(1) | **1.0** |
| viral infections | poliomyelitis | poliomyelitis; polioencephalitis | m | 2.3 | 4 days | disease-related | 2(2) | **0.0** |
| viral infections | poliomyelitis | status post poliomyelitis; hypoglycemia | f | 41 | n.a. | not disease-related | 2(2) | **0.0** |
| viral infections | poliomyelitis | status post poliomyelitis; heart attack | m | 61 | 53 years | not disease-related | 2(2) | **0.0** |
| viral infections | poliomyelitis | status post poliomyelitis | m | 66 | 28 years | n.a. | 3(3) | **0.0** |
|  |  |  |  |  |  |  |  |  |
| viral infections | viral meningitis / meningoencephalitis (not including HIV, measles virus, poliovirus or JC virus) with defined pathogen | HSV encephalitis; status post kidney transplantation | m | 64 | n.a. | disease-related | 2(2) | **0.0** |
| viral infections | viral meningitis / meningoencephalitis (not including HIV, measles virus, poliovirus or JC virus) with defined pathogen | HSV encephalitis | m | 63 | n.a. | disease-related | 3(3) | **3.0** |
| viral infections | viral meningitis / meningoencephalitis (not including HIV, measles virus, poliovirus or JC virus) with defined pathogen | HSV encephalitis | m | 67 | 7 days | disease-related | 2(2) | **3.0** |
| viral infections | viral meningitis / meningoencephalitis (not including HIV, measles virus, poliovirus or JC virus) with defined pathogen | HSV encephalitis | f | 14 | 3 weeks | disease-related | 2(2) | **1.0** |
| viral infections | viral meningitis / meningoencephalitis (not including HIV, measles virus, poliovirus or JC virus) with defined pathogen | HSV encephalitis | f | 42 | 23 days | disease-related | 1(1) | **3.0** |
| viral infections | viral meningitis / meningoencephalitis (not including HIV, measles virus, poliovirus or JC virus) with defined pathogen | CMV infection; HIV infection; pneumocystis carinii pneumonia; thrush | m | 38 | 7 months | disease-related | 2(2) | **3.0** |
| viral infections | viral meningitis / meningoencephalitis (not including HIV, measles virus, poliovirus or JC virus) with defined pathogen | CMV infection; HIV infection;  liver failure; acute cardiovascular failure | m | 39 | 1 year | n.a. | 2(2) | **0.5** |
| viral infections | viral meningitis / meningoencephalitis (not including HIV, measles virus, poliovirus or JC virus) with defined pathogen | HSV encephalitis | f | 0.4 | 6 days | disease-related | 2(2) | **1.0** |
|  |  |  |  |  |  |  |  |  |
| bacterial infections | tuberculosis | tuberculosis | f | 67 | n.a. | disease-related | 1(1) | **2.0** |
| bacterial infections | tuberculosis | tuberculosis | m | 68 | n.a. | disease-related | 1(1) | **2.0** |
| bacterial infections | tuberculosis | miliary tuberculosis; HIV | m | 32 | 10 months | disease-related | 1(1) | **2.0** |
| bacterial infections | tuberculosis | tuberculosis; bleed to death from duodenal ulcer | m | 55 | 2 months | not disease-related | 1(1) | **1.0** |
| bacterial infections | tuberculosis | tuberculosis | f | 4 | n.a. | disease-related | 3(2) | **2.7** |
| bacterial infections | tuberculosis | tuberculosis | m | 67 | 3 months | disease-related | 2(2) | **3.0** |
| bacterial infections | tuberculosis | tuberculosis | m | n.a. | n.a. | n.a. | 3(3) | **1.7** |
| bacterial infections | tuberculosis | tuberculosis | f | n.a. | n.a. | disease-related | 1(1) | **2.0** |
| bacterial infections | tuberculosis | tuberculosis | f | 83 | 5 weeks | disease-related | 1(1) | **1.0** |
| bacterial infections | tuberculosis | tuberculosis | m | 47 | 6 months | disease-related | 1(1) | **0.0** |
| bacterial infections | tuberculosis | tuberculosis | m | 64 | 4 weeks | disease-related | 2(2) | **2.0** |
| bacterial infections | tuberculosis | tuberculosis | f | 25 | 2 months | disease-related | 3(3) | **0.0** |
| bacterial infections | tuberculosis | tuberculosis | f | 41 | 4 weeks | disease-related | 2(2) | **2.5** |
|  |  |  |  |  |  |  |  |  |
| bacterial infections | syphilis | progressive paralysis | m | 60 | 7 years | disease-related | 6(6) | **0.3** |
| bacterial infections | syphilis | syphilis | m | n.a. | n.a. | n.a. | 5(5) | **0.6** |
| bacterial infections | syphilis | status post syphilis; acute cardiovascular failure | m | 80 | n.a. | not disease-related | 5(5) | **1.0** |
|  |  |  |  |  |  |  |  |  |
| bacterial infections | bacterial meningitis (not including tuberculosis or syphilis) with defined pathogen | Pneumococcal meningitis; pneumonia | f | 62 | 2 days | disease-related | 1(1) | **3.0** |
| bacterial infections | bacterial meningitis (not including tuberculosis or syphilis) with defined pathogen | Staphylococcus aureus meningoencephalitis; status post glioblastoma multiforme surgery | m | 43 | 3 weeks | disease-related | 2(2) | **3.0** |
| bacterial infections | bacterial meningitis (not including tuberculosis or syphilis) with defined pathogen | Staphylococcus aureus meningoencephalitis | m | 65 | 7 days | disease-related | 2(2) | **1.5** |
| bacterial infections | bacterial meningitis (not including tuberculosis or syphilis) with defined pathogen | Staphylococcus meningoencephalitis | f | 75 | 8 days | disease-related | 1(1) | **1.0** |
| bacterial infections | bacterial meningitis (not including tuberculosis or syphilis) with defined pathogen | Haemophilus influenzae meningitis | m | 2 | 2 days | disease-related | 3(3) | **3.0** |
| bacterial infections | bacterial meningitis (not including tuberculosis or syphilis) with defined pathogen | Klebsiella pneumoniae meningitis | m | 81 | n.a. | disease-related | 1(1) | **2.0** |
| bacterial infections | bacterial meningitis (not including tuberculosis or syphilis) with defined pathogen | Escherichia coli meningitis | m | 0.06 | n.a. | disease-related | 2(2) | **3.0** |
| bacterial infections | bacterial meningitis (not including tuberculosis or syphilis) with defined pathogen | Pseudomonas aeruginosa meningoencephalitis; pneumonia after renal transplantation | m | 25 | n.a. | disease-related | 2(2) | **2.0** |
| bacterial infections | bacterial meningitis (not including tuberculosis or syphilis) with defined pathogen | Streptococcus meningoencephalitis | m | 0.01 | 2 days | disease-related | 1(1) | **3.0** |
| bacterial infections | bacterial meningitis (not including tuberculosis or syphilis) with defined pathogen | Staphylococcus epidermidis meningoencephalitis | f | 78 | 1 week | disease-related | 2(2) | **2.0** |
| bacterial infections | bacterial meningitis (not including tuberculosis or syphilis) with defined pathogen | Haemophilus influenzae meningoencephalitis; otitis media | f | 0.17 | 3 days | disease-related | 2(2) | **3.0** |
| bacterial infections | bacterial meningitis (not including tuberculosis or syphilis) with defined pathogen | bacterial meningitis with proof of Enterococcus and Klebsiella pneumoniae | m | 53 | n.a. | disease-related | 1(1) | **2.0** |
|  |  |  |  |  |  |  |  |  |
| Inflammation/Infection without defined pathogen | acute lymphocytic meningitis/meningoencephalitis | lymphocytic meningoencephalitis; strangulation | f | 75 | n.a. | not disease-related | 1(1) | **0.0** |
| Inflammation/Infection without defined pathogen | acute lymphocytic meningitis/meningoencephalitis | temporal meningoecephalitis | m | 25 | 20 days | disease-related | 2(2) | **2.0** |
| Inflammation/Infection without defined pathogen | acute lymphocytic meningitis/meningoencephalitis | lymphocytic meningoencephalitis | f | 20 | 3 weeks | disease-related | 2(2) | **1.0** |
| Inflammation/Infection without defined pathogen | acute lymphocytic meningitis/meningoencephalitis | lymphocytic meningoencephalitis; gastroenteritis | m | 0.25 | 2 days | disease-related | 2(2) | **0.5** |
| Inflammation/Infection without defined pathogen | acute lymphocytic meningitis/meningoencephalitis | lymphocytic meningoencephalitis; acute respiratory disease | m | 1 | n.a. | n.a. | 1(1) | **2.0** |
| Inflammation/Infection without defined pathogen | acute lymphocytic meningitis/meningoencephalitis | lymphocytic meningoencephalitis | f | 53 | 6 days | disease-related | 4(4) | **0.8** |
| inflammation/Infection without defined pathogen | acute lymphocytic meningitis/meningoencephalitis | lymphocytic meningoencephalitis; renal failure; pneumonia | m | 69 | n.a. | not disease-related | 2(2) | **3.0** |
| inflammation/Infection without defined pathogen | acute lymphocytic meningitis/meningoencephalitis | lymphocytic meningoencephalitis; renal failure; pancreatitis | m | 41 | n.a. | not disease-related | 2(2) | **2.0** |
| inflammation/Infection without defined pathogen | acute lymphocytic meningitis/meningoencephalitis | lymphocytic meningoencephalitis; abdominal aortic aneurysm; pulmonary embolism; acute cardiovascular failure | m | 77 | n.a. | n.a. | 4(4) | **0.7** |
| inflammation/Infection without defined pathogen | acute lymphocytic meningitis/meningoencephalitis | lymphocytic meningoencephalitis; renal failure; coronary failure | m | 77 | n.a. | n.a. | 2(2) | **0.5** |
| inflammation/Infection without defined pathogen | acute lymphocytic meningitis/meningoencephalitis | lymphocytic meningoencephalitis | m | 47 | n.a. | n.a. | 2(2) | **0.0** |
| inflammation/Infection without defined pathogen | acute lymphocytic meningitis/meningoencephalitis | lymphocytic meningoencephalitis; heart attack | m | 45 | n.a. | not disease-related | 1(1) | **2.0** |
|  |  |  |  |  |  |  |  |  |
| inflammation/Infection without defined pathogen | acute granulocytic meningitis/meningoencephalitis | bacterial meningitis; kidney failure; gallbladder cancer | f | 69 | 1 day | disease-related | 1(1) | **2.0** |
| inflammation/Infection without defined pathogen | acute granulocytic meningitis/meningoencephalitis | acute bacterial meningitis | f | 20 | 2 days | disease-related | 2(2) | **2.0** |
| inflammation/Infection without defined pathogen | acute granulocytic meningitis/meningoencephalitis | basal meningitis after drainage of an abscess of the brain | m | 44 | 6 weeks | disease-related | 3(3) | **2.3** |
| inflammation/Infection without defined pathogen | acute granulocytic meningitis/meningoencephalitis | bacterial meningitis; bronchial carcinoma; respiratory failure | f | 47 | n.a. | not disease-related | 2(2) | **2.0** |
| inflammation/Infection without defined pathogen | acute granulocytic meningitis/meningoencephalitis | bacterial meningitis; bacterial hypophysitis; recurrent otitis media | f | 37 | n.a. | disease-related | 2(2) | **0.5** |
|  |  |  |  |  |  |  |  |  |
| metabolic disorders | central pontine myelinolysis | central pontine myelinolysis; sepsis | f | 65 | n.a. | not disease-related | 1(1) | **0.0** |
| metabolic disorders | central pontine myelinolysis | central pontine myelinolysis; sepsis;  multi-organ dysfunction | m | 55 | n.a. | n.a. | 1(1) | **0.0** |
| metabolic disorders | central pontine myelinolysis | central pontine myelinolysis; alcoholic cirrhosis | m | 31 | n.a. | n.a. | 3(3) | **0.0** |
| metabolic disorders | central pontine myelinolysis | central pontine myelinolysis; acute cardiovascular failure | m | 31 | n.a. | n.a. | 2(2) | **0.0** |
| metabolic disorders | central pontine myelinolysis | central pontine myelinolysis; bacterial meningitis | f | 58 | 13 days | disease-related | 3(3) | **2.3** |
| metabolic disorders | central pontine myelinolysis | alcoholic disease; electrolyte imbalance | m | 39 | 1 month | disease-related | 4(4) | **0.3** |
|  |  |  |  |  |  |  |  |  |
| autoimmune | multiple sclerosis | multiple sclerosis | f | 74 | Incidental | n.a. | 1(1) | n.a. |
| autoimmune | multiple sclerosis | multiple sclerosis | f | 71 | 20 | n.a. | 1(1) | n.a. |
| autoimmune | multiple sclerosis | multiple sclerosis | m | 49 | ? | n.a. | 1(1) | n.a. |
| autoimmune | multiple sclerosis | multiple sclerosis | m | 66 | ? | n.a. | 1(1) | n.a. |
| autoimmune | multiple sclerosis | multiple sclerosis | m | 38 | ? | n.a. | 1(1) | n.a. |
| autoimmune | multiple sclerosis | multiple sclerosis | m | 63 | Incidental | n.a. | 1(1) | n.a. |
| autoimmune | multiple sclerosis | multiple sclerosis | f | 66 | 30 | n.a. | 1(1) | n.a. |
| autoimmune | multiple sclerosis | multiple sclerosis (PP) | m | 74 | 10 | n.a. | 1(1) | n.a. |
| autoimmune | multiple sclerosis | multiple sclerosis (PP) | m | 57 | 15 | n.a. | 1(1) | n.a. |
| autoimmune | multiple sclerosis | multiple sclerosis (PP) | f | 45 | 14 | n.a. | 1(1) | n.a. |
| autoimmune | multiple sclerosis | multiple sclerosis (PP) | f | 70 | 7 | n.a. | 1(1) | n.a. |
| autoimmune | multiple sclerosis | multiple sclerosis (PP) | m | 57 | 11 | n.a. | 1(1) | n.a. |
| autoimmune | multiple sclerosis | multiple sclerosis (PP) | f | 61 | 19 | n.a. | 1(1) | n.a. |
| autoimmune | multiple sclerosis | multiple sclerosis (PP) | m | 51 | 8 | n.a. | 1(1) | n.a. |
| autoimmune | multiple sclerosis | multiple sclerosis (PP) | f | 60 | 14 | n.a. | 1(1) | n.a. |
| autoimmune | multiple sclerosis | multiple sclerosis (PP(?)) | f | 59 | 9 | n.a. | 1(1) | n.a. |
| autoimmune | multiple sclerosis | multiple sclerosis (PP(?)) | m | 60 | 10 | n.a. | 1(1) | n.a. |
| autoimmune | multiple sclerosis | multiple sclerosis (PP(?)) | f | 52 | 8 | n.a. | 1(1) | n.a. |
| autoimmune | multiple sclerosis | multiple sclerosis (RR) | f | 35 | 23 | n.a. | 1(1) | n.a. |
| autoimmune | multiple sclerosis | multiple sclerosis (SP) | f | 28 | 4 | n.a. | 1(1) | n.a. |
| autoimmune | multiple sclerosis | multiple sclerosis (SP) | m | 69 | 7 | n.a. | 1(1) | n.a. |
| autoimmune | multiple sclerosis | multiple sclerosis (SP) | m | 54 | 11 | n.a. | 1(1) | n.a. |
| autoimmune | multiple sclerosis | multiple sclerosis (SP) | m | 70 | 32 | n.a. | 1(1) | n.a. |
| autoimmune | multiple sclerosis | multiple sclerosis (SP) | m | 52 | 16 | n.a. | 1(1) | n.a. |
| autoimmune | multiple sclerosis | multiple sclerosis (SP) | m | 63 | 19 | n.a. | 1(1) | n.a. |
| autoimmune | multiple sclerosis | multiple sclerosis (SP) | m | 57 | 12 | n.a. | 1(1) | n.a. |
| autoimmune | multiple sclerosis | multiple sclerosis (SP) | f | 53 | 25(?) | n.a. | 1(1) | n.a. |
| autoimmune | multiple sclerosis | multiple sclerosis (SP) | f | 81 | 36 | n.a. | 1(1) | n.a. |
| autoimmune | multiple sclerosis | multiple sclerosis (SP(?)) | m | 61 | 36 | n.a. | 1(1) | n.a. |
| autoimmune | multiple sclerosis | multiple sclerosis (SP(?)) | m | 66 | 44 | n.a. | 1(1) | n.a. |
| autoimmune | multiple sclerosis | multiple sclerosis (SP(?)) | m | 71 | ? | n.a. | 1(1) | n.a. |
| autoimmune | multiple sclerosis | multiple sclerosis (SP(?)) | f | 59 | 16 | n.a. | 1(1) | n.a. |
| autoimmune | multiple sclerosis | multiple sclerosis (SP(?)) | f | 60 | 25 | n.a. | 1(1) | n.a. |
| autoimmune | multiple sclerosis | multiple sclerosis |  | 74 | Incidental | n.a. | 1(1) | n.a. |
| ^*^ one PML-Biopsy-case was included in the table  ^1^ assessment of meningeal infiltration  0= no infiltration 1= sparse cellular infiltration (less than 50 infiltrating cells per high power field) 2= moderate cellular infiltration (more than 50 and less than 200 infiltrating cells per high power field) 3= dense cellular infiltration (more than 200 infiltrating cells per high power field)  PP= primary progressive disease course, RR= relapsing remitting disease course, SP= secondary progressive disease course  n.a. = not available | | | | | | | | |
